# A flexible triboelectric-piezoelectric hybrid nanogenerator based on P(VDF-TrFE) nanofibers and PDMS/MWCNT for wearable devices

**DOI:** 10.1038/srep36409

**Published:** 2016-11-02

**Authors:** Xingzhao Wang, Bin Yang, Jingquan Liu, Yanbo Zhu, Chunsheng Yang, Qing He

**Affiliations:** 1 National Key Laboratory of Science and Technology on Micro/Nano Fabrication, Department of Micro/Nano Electronics, Shanghai Jiao Tong University, Shanghai, 200240, China; 2 School of Biomedical Engineering, Shanghai Jiao Tong University, Shanghai, 200240, China

## Abstract

This paper studied and realized a flexible nanogenerator based on P(VDF-TrFE) nanofibers and PDMS/MWCNT thin composite membrane, which worked under triboelectric and piezoelectric hybrid mechanisms. The P(VDF-TrFE) nanofibers as a piezoelectric functional layer and a triboelectric friction layer are formed by electrospinning process. In order to improve the performance of triboelectric nanogenerator, the multiwall carbon nanotubes (MWCNT) is doped into PDMS patterned films as the other flexible friction layer to increase the initial capacitance. The flexible nanogenerator is fabricated by low cost MEMS processes. Its output performance is characterized in detail and structural optimization is performed. The device’s output peak-peak voltage, power and power density under triboelectric mechanism are 25 V, 98.56 μW and 1.98 mW/cm^3^ under the pressure force of 5 N, respectively. The output peak-peak voltage, power and power density under piezoelectric working principle are 2.5 V, 9.74 μW, and 0.689 mW/cm^3^ under the same condition, respectively. We believe that the proposed flexible, biocompatible, lightweight, low cost nanogenerator will supply effective power energy sustainably for wearable devices in practical applications.

With the growing development of wearable and implantable devices, nanogenerators (NGs) have been paid considerable attention in recent years due to their high conversion efficiency and low-cost with organic nanomaterials^1^,^2^,^3^. The powers of micro/nano scale devices or systems supplied by traditional batteries have some problems such as miniaturization, compatibility and long lifetime^4^,^5^. Thus harvesting the energy from environments by NGs can be a potential and effective alternative to meet such requirements. The traditional mechanisms of NGs have been reviewed^6^,^7^,^8^, including electrostatic^9^,^10^, piezoelectric^11^,^12^ and electromagnetic^13^,^14^ methods. However, those devices usually cannot satisfy the need of power supply for electronic devices due to their complex fabrication processes, low output power and biocompatibility. Moreover, most of devices work under single mechanism and their output performance is actually limited in service.

Due to relatively high output and easy fabrication process, triboelectric nanogenerators (TENGs) appeared recently and are noticeable as an energy scavenging method^15^,^16^. They generate positive and negative charges on two dissimilar surfaces when the two layers contact and separate each other. Various types materials are deployed as dissimilar surfaces such as Polydimethylsiloxance (PDMS)^17^,^18^, Kapton^19^, ethylene-vinyl acetate copolymer (EVA)^20^, Polyethylene terephthalate (PET)^21^, Perfluoroalkoxy (PFA)^22^, Nylon^23^, Polyvinylchloride^24^, Polytetrafluoroethylene (PTFE)^25^,^26^,^27^,^28^,^29^,^30^ and metal materials^31^. According to the triboelectric series, the larger affinity difference between two different materials contributes to the higher output performance^32^. Among them, PDMS and PET film as friction layers are reported to generate high output power from body movements^33^,^34^. Cu and polyolefin as triboelectric materials was proposed in a triboelectric generator to self-power instantaneous tactile imaging^35^. Zhang *et al*.^36^ proposed an electrode-free triboelectric generator which harvested human body motions using EVA material. Lee *et al*.^37^ demonstrated a skin based finger motion sensor under triboelectric effect which can measure the static and dynamic positions of fingers. Meanwhile, piezoelectric polymers of poly(vinylidenefluoride) (PVDF) and its copolymer poly(vinylidenefluoride-co-trifluoroethylene) (P(VDF-TrFE)) have been previously investigated in piezoelectric nanogenerators (PENGs)^38^,^39^ owing to their flexibility and processing simplicity. PVDF film was proposed as a functional piezoelectric material of a flexible curved generator which converted mechanical energy to electricity from body movements^40^. P(VDF-TrFE) thin film fabricated by spin-coating process for NG can exhibit the open-circuit voltage of 7 V and current density of 0.56 μA/cm^3^^41^. Currently, most of NGs have been reported to harvest mechanical energy by single mechanism. Therefore, it would be highly desirable to employ multi mechanisms at the same time not only to miniaturize NGs but also to obtain high output performance for powering the devices. For example, a hybrid NG based on piezoelectric and pyroelectric mechanisms based was proposed to harvest thermal and mechanical energy using PVDF film^42^,^43^. A highly stretchable piezoelectric and pyroelectric hybrid NG was fabricated, where P(VDF-TrFE) film was spin-coating and graphene was top flexible electrode^44^.

In this work, we present a stretchable, flexible triboelectric and piezoelectric nanogenerator (TPENG) based on P(VDF-TrFE) nanofibers to further increase the output power. Further, we dispersed multi-wall carbon nanotubes (MWCNTs) into PDMS to obtain lower internal resistance of this generator. Meantime, small pillars are fabricated on the surface of PDMS/MWCNT membrane for increasing the roughness and enhance the triboelectric performance. P(VDF-TrFE) nanofibes-based film fabricated by electrospinning process is applied as functional piezoelectric layer, and its output power from piezoelectric mechanism would further increase the total output power. Moreover, through optimization processes and characterization, the presented TPENG has been used for the application of finger motion detector.

## Results

### Structure and working principle

**Figure 1:**
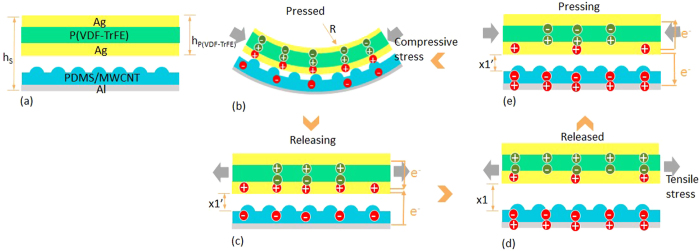
Structure and working principle of the triboelectric and piezoelectric hybrid nanogenerator. (**a**) The cross-section structural schematic; (**b**–**e**) Charge distribution of triboelectric and piezoelectric effect during force pressing and releasing. Inside of the picture, 0 < *x*_*1*_′ < *x*_*1*_, *h*_*s*_ is the summation height of TPENG, *h*_*P(VDF-TrFE)*_ is the height of P(VDF-TrFE) film, *R* is the stretching radius.

The TPENG is composed of two films, which include a bottom friction layer of PDMS/MWCNT membrane with patterned micro structure and a top functional piezoelectric layer of P(VDF-TrFE) nanofibers with double layers electrodes. There is a small separation distance of 300 μm between two friction layers to insulate each other. The schematic of TPENG is shown in Fig. 1(a). In the steady equilibrium state, micro-patterned PDMS/MWCNT membrane and P(VDF-TrFE) film separate and there’s no charged electrons. When an external force is applied on the device, like fingertip force, they are brought into contact and the device is deformed. At first, piezoelectric functional layer is subjected to compressive stresses, resulting in positive and negative electrons on the electrodes. Meanwhile, the contacted surface is charged based on triboelectric effect, as shown in Fig. 1(b). With the releasing continually, PDMS/MWCNT membrane starts to separate with electrode along with the gap of *x*_*1*_′, as shown in Fig. 1(c). During the releasing process, P(VDF-TrFE) nanofibers-based film recovers original state and it is subjected to tensile stress. Accordingly, the electric potential difference leads to the output electrons flowing in the circuit between two friction layers. When TPENG fully releases to its original state, the gap of *x*_*1*_ exists to maintain the stable state of electrical flowing, as shown in Fig. 1(d). However, if the press force is applied again, this gap decreases and causes reverse orientation electric current, as shown in Fig. 1(e). Subsequently, the external pressure operates and piezoelectric functional layer deforms. The occupied compressive force leads electrons flowing on the opposite direction in piezoelectric film. At this stage, a working cycle is completed and electric energy generation process is occurred^45^.

**Figure 2:**
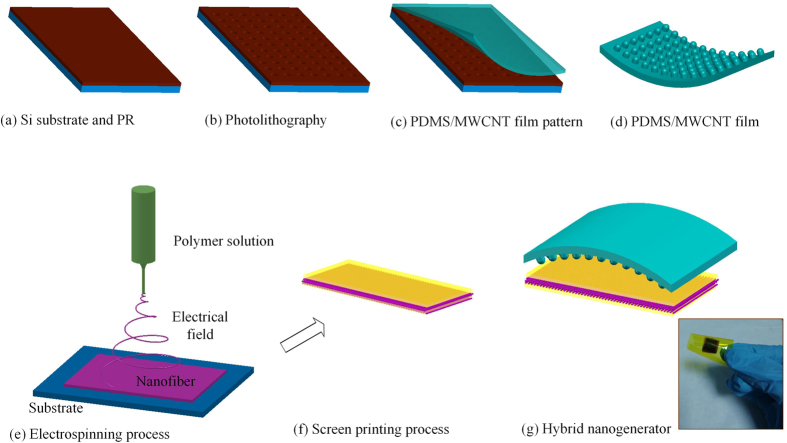
Structure and the fabrication process of the triboelectric and piezoelectric hybrid nanogenerator. (**a**–**d**) The fabrication flowchart of the PDMS/MWCNT friction layer; (**e**–**f**) The electrospinning process of P(VDF-TrFE) nanofiber; (**g**) The fabricated hybrid nanogenerator. Attached photo shows the flexible TPENG which was packaged with PI film.

Here, during single cycle, we define a hybrid NG with the parallel plate contact-mode. Therefore, the triboelectric voltage can be calculated according to Eq. (1)^46^,


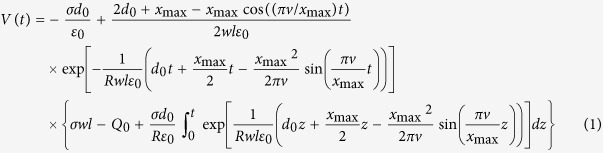


where *σ* is the tribo-charge surface density, *d*_*0*_ is the effective dielectric thickness of NG 
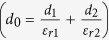
, *d*_*1*_ and *d*_*2*_ are the dielectric thickness of two triboelectric layers, respectively, *ε*_*0*_ is permittivity, *x*_*max*_ is the maximum separation distance between two parallel plate (*x*_*max*_ is *x*_*1*_ in the Fig. 2(f)), *v* is the velocity of moving layer, *w* is the width, *l* is the length, *R* is the internal resistance, *Q*_*0*_ is the initial charges on the plates. To enhance the performance and make a high-output NG, the appropriate roughness on the surface of triboelectric materials is needed.

Additionally, the piezoelectric voltage of the NG can be expressed as Eq. (2)^41^


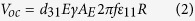


where *d*_*31*_ is the piezoelectric efficiency; *E* (4–6 GPa) is the Young’s modulus of P(VDF-TrFE); *γ* is a geometry effect parameter, which is introduced to evaluate the fringe effect with *A*_*E*_′ = *γA*_*E*_; *A*_*E*_ is the effective working area of the flexible piezoelectric thin film, *A*_*E*_′ is the effective poling area^47^; *f* is the working frequency, *ε*_*11*_ is the normal strain along *x*_*1*_ direction with stretching radius *R*, 
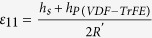
; *R*′ is the inner resistance of P(VDF = TrFE) film (*R, h*_*s*_, *h*_*P(VDF-TrFE)*_ are shown in Fig. 1).

### Material and Improvement

In order to decrease the NG’s internal resistance, we dispersed multi-wall carbon nanotubes (MWCNTs, XFM07, Nanjing XFNANO Materials Tech Co., Ltd) into PDMS (Sylgard 184 A, Dow Corning Company) to fabricate the friction membrane of TPENG. According to our early research, the PDMS/MWCNT composite film has much higher dielectric parameter than the pure PDMS film under low function frequency. The dispersion of MWCNT is a key factor that influences the conductivity of the PDMS film^48^. The fabrication process of PMDS/MWCNT composite membrane is shown in Fig. 2(a–d). The micro patterned structures on the surface should be formed to increase the roughness of composite membrane to improve the output power of triboelectric NG. The height of cylinder is controlled by the thickness of photoresist. To achieve a flexible TPENG, the organic P(VDF-TrFE) material is selected for piezoelectric layer due to its high flexibility, excellent mechanical and chemical stability compared to inorganic piezoelectric materials. Meanwhile, electrospinning process has the advantage that provides high bias electric field (usually from 10 kV ~ 80 kV), which can directly array the electric dipole along the major axis direction of the fiber^49^,^50^,^51^. Therefore, part of α crystal phase inside of P(VDF-TrFE) nanofiber converses into β crystal phase. Then the PVDF-TrFE nanofibers can generate electric current under external pressure. The electrospinning process was shown in Fig. 2(e–f). The thickness of electrode is about 10 μm. The dimension of each layer is 8 mm × 8 mm. The thickness of P(VDF-TrFE) nanofiber and PDMS-MWCNT film is 85 μm and 45 μm, respectively. The surface area and volume of this device is 0.64 cm^2^ and 0.0128 cm^3^, respectively. Compared to the previously reported devices^52^,^53^,^54^, this device has good flexibility and stretch ability as shown in Fig. 2(g) due to thin thickness and used flexible materials.

**Figure 3:**
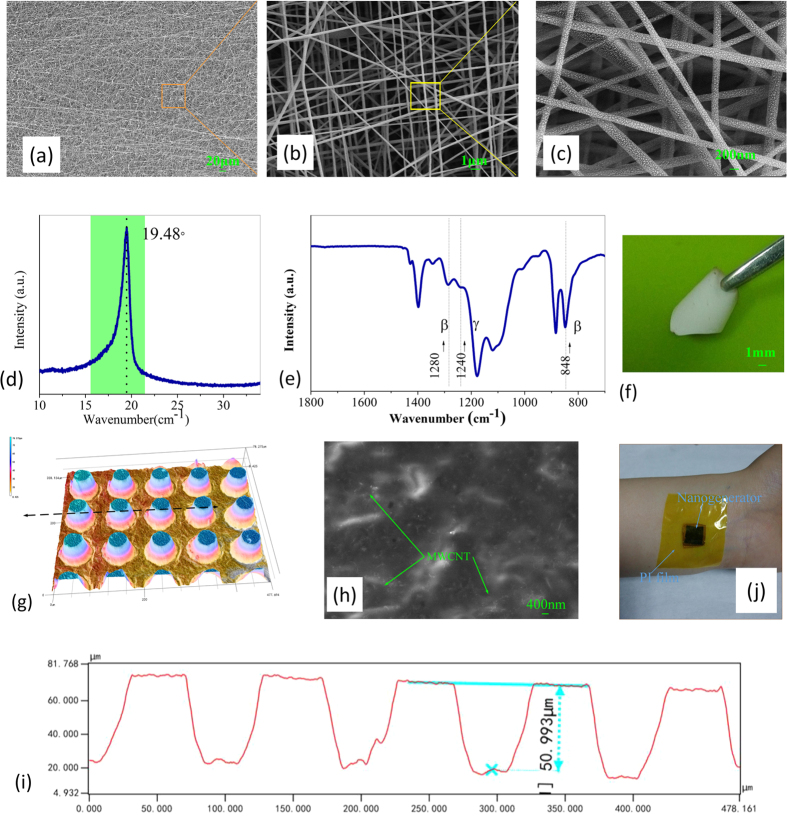
(**a**–**c**) SEM picture of P(VDF-TrFE) nanofibers. (**d**) XRD patterns and (**e**) FTIR spectra of the P(VDF-TrFE) nanofibers. (**f**) Physical diagram of P(VDF-TrFE) nanofiber. (**g**) 3-D diagram of the micro-patterned PDMS/MWCNT friction surface. (**h**) SEM images of MWCNTs under higher magnitude. (i) Cross-section measurement of micro-patterned PDMS/MWCNT membrane where is located on the position pointed in Fig. 3(g,j) TPENG coated with PI film and attached to human skin.

The SEM images of electrospinning P(VDF-TrFE) nanofibers are shown in Fig. 3(a–c) with different enlarged amplitudes. The average diameter of nanofibers is 200 ± 20 nm. Obviously, uniform slender nanofibers with disordered distribution are been fabricated by electrospinning process. As shown in Fig. 3(d,e), the nature of the polymer crystalline phase presented in nanofibers can be identified by XRD and FTIR, respectively. The XRD curve shows a dominant diffraction peak corresponding to typical *β* crystal phase peak with around 2*θ* = 19.48°. And the vibration bands at 848 cm^−1^ and 1280 cm^−1^are assigned to the absorption bands of the *β* crystal phase. The vibration brand at 1240 cm^−1^ is assigned to the absorption band of the *γ* crystal phase. Based on these results, it’s significant that we achieve uniform nanofibers which are accounted for most of the typical *β* crystal phase inside^55^,^56^. Obtained P(VDF-TrFE) nanofiber demonstrates a good flexibility and uniformity as expected, as shown in Fig. 3(f).

By the process of the viscous PDMS/MWCNT precursor, the micro circular truncated cone pillars are achieved and formed to increase the reliability and improve the output performance of generator. To investigate the morphology of PDMS/MWCNT membrane, we experimentally obtain the profile of the microstructure using 3D profilometer (KEYENCE VK-X Series), as shown in Fig. 3(g,i). Obviously, the pillars are regularly arrayed and different colors represent different values of height. They have uniform height, gap and diameter of 50 μm, and sculptured surfaces on its top and gap. It can be concluded that the MWCNTs are uniformly dispersed in PDMS from enlarged SEM photograph, as shown in Fig. 3(h). To perform characterization, TPENG was assembled and coated with PI film as its packaging^57^. TPENG was attached to skin as shown in Fig. 3(j) and can be a potential power source for wearable devices or systems.

### Output performance

**Figure 4:**
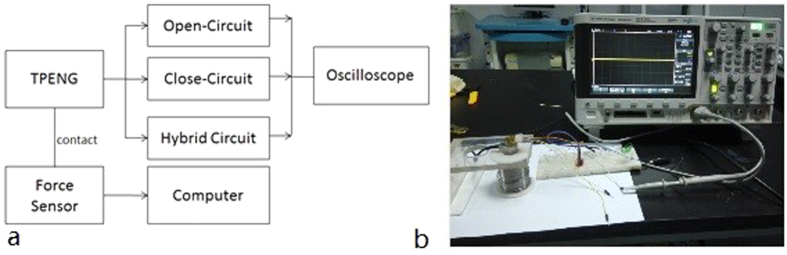
(**a**) The schematic photo of experimental platform for the characterization of nanogenerator. (**b**) Testing platform of TPENG.

**Figure 5:**
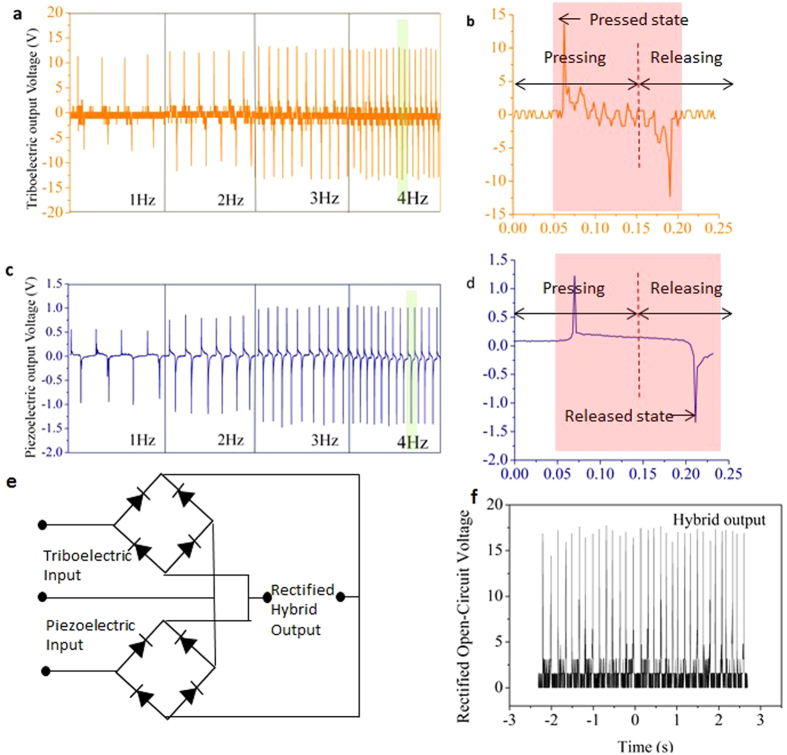
(**a**) The open-circuit performance of the triboelectric under the force of 5 N as a function of frequency. (**b**) The magnified view of data corresponding to 4 Hz triboelectric results (green area on the left). (**c**) The open-circuit performance of the piezoelectric under the force of 5 N as a function of frequency. (**d**) The magnified view of data corresponding to 4 Hz piezoelectric results (green area on the left). (**e**) The circuit diagram of the hybrid output which piezoelectric and triboelectric outputs are combined in parallel. (**f**) Hybrid open-circuit output voltage.

We characterized the TPENG’s output performance under varied frequencies from 1 to 4 Hz based on the designed experiment platform (Fig. 4). The open-circuit triboelectric and piezoelectric output voltages of NG are shown in the Fig. 5(a,c), respectively. With the increasing frequency of the external force, the peak to peak triboelectric voltage increased from 17.5 V at 1 Hz to 25 V at 3 Hz under 5 N pressure forces, and the peak to peak piezoelectric voltage increased from 1.5 V at 1 Hz to 2.5 V at 3 Hz. This is because the charge transfer is maintained equal under different frequencies, but the output increment is caused by faster electron flow at low frequencies (i.e. 1–4 Hz)^58^. The working process of the hybrid NG in one cycle is explained in the Fig. 5(b,d), corresponding to each state of piezoelectric and triboelectric output voltages, respectively. The triboelectric output voltage is obviously much higher and sharper than that of piezoelectric NG, and there is a delay in the releasing part. This is because the residual strain in the piezoelectric film is maintained and triboelectric charges are compensated faster than piezoelectric one^59^. Triboelectric and piezoelectric output voltages are kept at 25.0 V and 2.5 V under 4 Hz because the external electrons flowing to reach equilibrium^21^. Full-wave bridge diodes and the parallel connection of piezoelectric and triboelectric are utilized as shown in Fig. 5(e). The rectified hybrid open-circuit output voltage was shown in Fig. 5(f). Its average peak output voltage was about 17 V.

**Figure 6:**
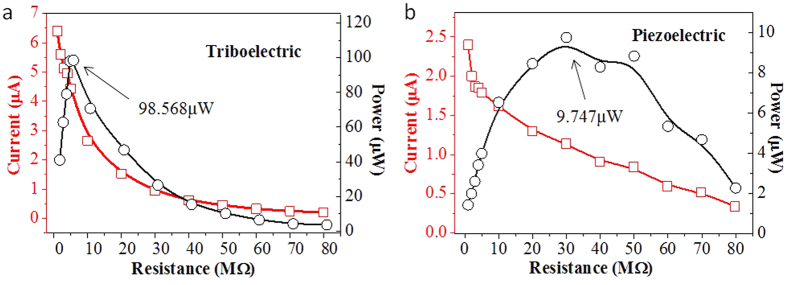
(**a**) The close-circuit triboelectric current and power under varied external loading resistances. (**b**) The close-circuit piezoelectric current and power under varied external loading resistances.

The output current and power of the device in close-circuit under various loading resistance ranged from 1 Ω to 80 MΩ are characterized, as shown in Fig. 6. It is seen that the corresponding triboelectric maximum power is 98.56 μW at 5 MΩ and piezoelectric maximum power is 9.747 μW at 30 MΩ. It demonstrates that the optimum loading matched resistances are different for triboelectric and piezoelectric mechanisms. It is obtained that the optimized resistances can be controlled by the MWCNT doping concentration for the practical application based on this proposed concept. The instantaneous triboelectric and piezoelectric output power densities are calculated as 1.98 mW/cm^3^ and 0.689 mW/cm^3^, respectively.

## Discussion

**Figure 7:**
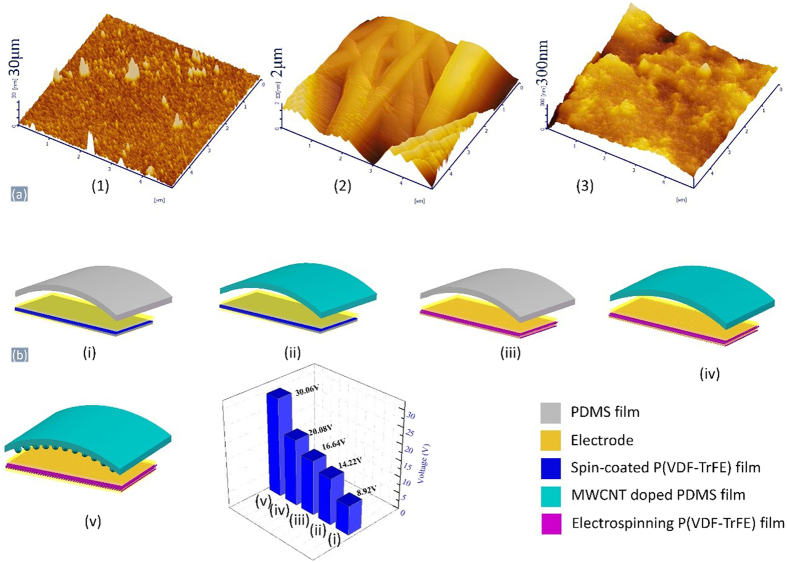
(**a**) The AFM images of different films surface: (1) Spin-coated P(VDF-TrFE) film with vapored electrodes; (2) Electrospinning P(VDF-TrFE) film with vapored electrodes; (3) Electrospinning P(VDF-TrFE) film with screen-printed electrodes. (**b**) Structures and performance comparison of five types of nanogenerators: (i) The combination of spin-coated P(VDF-TrFE) film with vapored electrodes and pure PDMS film; (ii) The combination of spin-coated P(VDF-TrFE) film with vapored electrodes and PDMS/MWCNT composite film; (iii) The combination of electrospun P(VDF-TrFE) film with screen-printed electrodes and pure PDMS film; (iv) The combination of electrospun P(VDF-TrFE) film with screen-printed electrodes and PDMS/MWCNT composite film; (v) The combination of electrospun P(VDF-TrFE) film with screen-printed electrodes and PDMS/MWCNT composite film; (vi) The results of combinations.

As mentioned before that the roughness of triboelectric friction layer strongly affected output performance of TENG. It is described that the TENG would achieve ideal and optimum output performance when the friction layers had micro or nano scale structures. Here, we prepared different friction layer structures to investigate the outputs of TPENG. The spin-coated P(VDF-TrFE) film with vapored electrodes, electrospinning film with vapored electrodes and electrospinning film with screen-printed electrodes are fabricated and their AFM surface images are shown in Fig. 7a(1–3), respectively. Spin-coated P(VDF-TrFE) film has relatively lower roughness of 30 nm. While electrospinning film with vapored electrodes has surface roughness of 2000 nm and screen-printed one has 800 nm. Meantime, five types of the other friction layers are produced, including spin-coated PDMS film, PDMS film with micro structures, spin-coated PDMS/MWCNT film and PDMS/MWCNT film with micro structures. Their combinations are shown in Fig. 7b(i–v) and their triboelectric output voltages of NG under the pressure of 5 N and the frequency of 5 Hz are shown in Fig. 7b(vi). Their corresponding voltages are 8.92 V, 14.22 V, 16.64 V, 20.08 V and 30.06 V, respectively. By comparison, it can conclude that PDMS/MWCNT film has better output performance than PDMS film due to faster electron flowing of MWCNTs doping, and micro structures also contributes to increase the output voltages. Meantime, the optimum property happened to the combination of PDMS/MWCNT film with micro structures and electrospinning P(VDF-TrFE) film with screen-printed electrodes.

**Figure 8:**
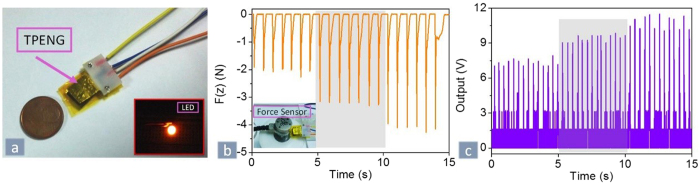
(**a**) Photograph of the fabricated TPENG which can lighten one LED bulb during the moment of being lit up by mechanical force of finger. (**b**) Applied force was recorded by force sensor when the finger forces of 2 N, 3 N and 4 N are pressed on TPENG. (**c**) The output voltage of TPENG under the varied forces of 2 N, 3 N and 4 N.

Due to the flexibility of fabricated TPENG, the full-contact state can be easily achieved by fingertip pressing. Thus we utilized it to monitor fingertip motion. The proposed device is shown in Fig. 8(a) and it can light up a commercial LED bulb when the force is applied by finger (snapshot of LED shown in Fig. 8(a)). When the finger forces are varied from 2 N to 4 N, as shown in Fig. 8(b), the corresponding output voltages of TPENG are increased from 7 V to 11 V, as shown in Fig. 8(c). It can also be used as a finger-motion sensor. The proposed hybrid NG has some advantages: a) it produces relatively high output power in small scale matched with human fingertip force, which is capable of lighting LED; b) it can be easily fabricated with lower cost and achieved with flexibility and biocompatibility; c) it utilizes PDMS/MWCNT composite membrane not only to tune the internal resistance of device but also to combine with P(VDF-TrFE) nanofiber to achieve a higher output performance for matching the loading resistance. Compared with the other NGs mentioned above, the achieved hybrid NG is superior in terms of output voltage.

In summary, a flexible triboelectric and piezoelectric hybrid NG is proposed and fabricated, which is composed of PDMS/MWCNT membrane, P(VDF-TrFE) nanofibers by electrospinning method, and electrodes by screen-printing process. It is observed that the output performance of TPENG will be improved by MWCNTs doped PDMS membrane as well as patterned pillars micro-structure. Meantime, as an optimum proposal, electrospinning P(VDF-TrFE) nanofibers film with screen-printed electrodes will achieve higher outpower. In the open-circuit, the triboelectric and piezoelectric output peak voltages of TPENG are 25 V and 2.5 V under the pressure of 5 N, respectively. While connecting with vaired external resistances, the triboelectric output power and power density are 98.56 μW and 1.98 mW/cm^3^ under the matched resistance of 5 MΩ, and the piezoelectric output power and power density are 9.747 μW and 0.689 mW/cm^3^ under the matched resistance of 30 MΩ. This proposed TPENG will be a small-scale, flexible and apposite power promising source for wearable and implantable devices and portable electronic devices.

## Methods

### P(VDF-TrFE) nanofiber

The syringe filled with P(VDF-TrFE) solution is connected with a high positive voltage of 10 kV. In the precursor solution, P(VDF-TrFE) (volume ratio of 55/45) is dissolved in N-N dimethylformamide (DMF) solution, and the weight percentage is 15%. And to get electrospinning solution, 55 wt% acetone is dissolved in the precursor solution to realize good volatility. A grounded collector with metal surface electrode is placed 10 cm away from the needle to gather the nanofibers. The syringe pump injection has a uniform speed of 0.4 ml/h. The fabricated nanofibers are annealed under 85 °C in vacuum. In order to capture electrons from piezoelectric layer under deformation, conductive silver paste is covered on both sides by screen printing method.

### PDMS/MWCNT composite membrane

PDMS solution and MWCNT are mixed in the weight ratio of 10:1. Toluene is dissolved in the mixture to disperse MWCNT uniform distribution for 24 hours by ultrasonic method. The cylinder array with the diameter of 50 μm is patterned by photolithography. Then the mixture of PDMS/MWCNT is coated, pressed and peeled after a thermal curing process under 80 °C.

### Method of output performance testing

To explore the triboelectric and piezoelectric performance of the TPENG, the experiment platform is set up, as shown in Fig. 4. It consists of a 3-axial force pressure sensor (ATI, NANO17), an oscilloscope (Agilent 2000X) and a testing circuit. The circuit is connected with double silver electrodes of P(VDF-TrFE) film to characterize the output piezoelectric performance. While this circuit is connected with the bottom silver electrode and PDMS/MWCNT film, it can show the output performance of triboelectric mechanism. At the same time, the TPENG is applied by fingertip force and the force is monitored by the pressure sensor. It could directly monitor the pressure values from *x, y, z* direction at the same time and record the force data in real time. Thus a continuous and stable fingertip force will be obtained. Besides, the output voltages in open and close circuit are measured from the oscilloscope.

## Additional Information

**How to cite this article**: Wang, X. *et al*. A flexible triboelectric-piezoelectric hybrid nanogenerator based on P(VDF-TrFE) nanofibers and PDMS/MWCNT for wearable devices. *Sci. Rep.* **6**, 36409; doi: 10.1038/srep36409 (2016).

**Publisher’s note:** Springer Nature remains neutral with regard to jurisdictional claims in published maps and institutional affiliations.
